# Swimming behavior of *Daphnia dentifera* is influenced by *Australozyma monospora* infection

**DOI:** 10.1371/journal.pone.0346784

**Published:** 2026-05-11

**Authors:** Jeannette E. Cullum, Vanessa Rivera-Quiñones, Ilona A. Menel, Zoi Rapti, Carla E. Cáceres

**Affiliations:** 1 Program in Ecology, Evolution, and Conservation Biology, University of Illinois Urbana - Champaign, Urbana, Illinois, United States of America; 2 Carl R. Woese Institute for Genomic Biology, University of Illinois Urbana - Champaign, Urbana, Illinois, United States of America; 3 Department of Mathematics, University of Illinois Urbana - Champaign, Urbana, Illinois, United States of America; 4 Department of Evolution, Ecology, and Behavior, School of Integrative Biology, University of Illinois Urbana - Champaign, Urbana, Illinois, United States of America; Central University of South Bihar, INDIA

## Abstract

By changing the behavior of infected hosts, pathogens can shape the outcome of both disease transmission and other interspecific interactions within the food web. This change in behavior is most often recognized in trophically-transmitted parasites, where changing the behavior of an intermediate host can increase the rate at which the parasite is transmitted to the next host. However, behavioral changes also occur in terminal hosts. In a laboratory experiment, we investigated if infection by the ascomycete yeast *Australozyma monospora* (formerly *Metschnikowia bicuspidata*) and/or the time since infection changes the swimming behavior over time of its freshwater cladoceran host, *Daphnia dentifera*. Infection influenced the net distance and total distance traveled as well as the swimming speed and mean depth of the *Daphnia*. Uninfected individuals traveled further, both in terms of net distance and total distance, and they also swam faster. The influence of time since infection was more nuanced. Swimming speed, net distance, and total distance traveled varied with time, but mean depth did not. Though we found no main effect of time since infection on mean swimming depth, time did interact with infection status to influence mean depth. A better understanding of how pathogens may alter the movement and habitat selection of infected hosts can help to inform predictive models of disease spread in lakes that are ever-changing due to increased anthropogenic stressors.

## Introduction

Parasites and pathogens can modify the behavior of their host. The most recognizable examples involve trophic-transmission [1–3]. Here, the fitness benefit to the parasite is clear; by altering the behavior of a host, the parasite is more likely to encounter the host needed to complete its life cycle or a favorable habitat for a free-living stage. Even in single-host systems, however, changes in host behavior occur and can influence transmission. For example, behavioral changes in movement or habitat selection may change contact rates between infected and susceptible hosts and/or contact rates between susceptible hosts and free-living infective propagules [4,5]. It can also influence predation risk affecting transmission [6–8]. These behavioral changes may be a direct manipulation (e.g., of the central nervous system) or an indirect consequence of reducing the energy the host has to allocate to particular behaviors [3,9,10]. Given the complexity of effects that determine transmission, understanding when modifications to host behavior occur, whether they occur due to direct manipulation or reduced energy, and the direct and indirect effects of these modifications on transmission is needed to effectively predict the timing and severity of disease outbreaks in natural populations.

Many pathogens in aquatic systems have a free-living stage where changes in the swimming behavior of their hosts can alter disease transmission through multiple mechanisms [11,12]. First, because infective propagules of such pathogens are often unevenly distributed in the water column, changes in host swimming behavior may alter the host-pathogen encounter rates [12–14]. Second, predation risk varies vertically in aquatic environments; for systems in which predation influences transmission, host swimming behavior modifies transmission via its effects on encounter with predators [6–8]. Third, transmission can be altered by host resistance and recovery through mechanisms involving both temperature and resources, which often also vary in both their vertical and horizontal distribution in aquatic environments [15–17].

Several previous studies have documented the importance of swimming behavior in aquatic host-pathogen systems. Decaestecker and colleagues demonstrated that depth selection (a function of swimming behavior) in *Daphnia magna* Straus, 1820 [18] modulated infection risk with the bacterium *Pasteuria ramosa* Metschnikoff, 1888 [19] at the cost of increased predation risk [20]. This work suggests several fitness trade-offs where individuals must select habitats to facilitate predator and pathogen evasion while maximizing egg development time and resource acquisition. Other work has shown that multiple pathogens alter *Daphnia magna* depth selection resulting in infected individuals being observed at greater depths during daytime conditions than uninfected individuals [21]. Similarly, sampling of a Wisconsin lake revealed an inhibition of the day versus night depth selection behavior (i.e., diel vertical migration, or DVM) in *Daphnia pulicaria* Forbes, 1893 [22] infected with a chytridiomycete, where the infection intensity predicted the degree of DVM inhibition [23]. These field and laboratory studies demonstrate that swimming behavior plays an important role in a host’s encounter rate with a pathogen and resource acquisition which in turn affects pathogen growth and the spread of disease. They additionally demonstrate that both infection itself and the intensity of infection can alter host swimming behavior, which they found reflected in modified depth selection behavior.

*Daphnia dentifera* Forbes, 1893 [22] (then distinguished from the closely related species *Daphnia galeata* Sars, 1864 [24]), a common species of freshwater zooplankton in the midwestern United States, is a known host for the ascomycete *Australozyma monospora* (formerly *Metschnikowia bicuspidata* (Metschnikoff) Kamieński, 1899 [25–29]). Fungal spores are ingested by the host while filter-feeding, and infection is established when spores penetrate the gut, avoid the host immune system, and reproduce [28,29]. Late-stage infections are easily diagnosed by visual inspection and spores are returned to the water column following the death of the host. We asked if infection by *Australozyma monospora* can alter swimming behavior in *Daphnia dentifera.* In a series of laboratory trials, we measured the average depth in the water column, distance traveled, net distance traveled, and swimming speed in five host genotypes. Based on previous work with other *Daphnia* species, we predicted that we would find infected individuals deeper in the water column, and that they would travel less and move more slowly. We also predicted that as the time since infection increased, we would see larger differences in the behavioral responses between infected and uninfected individuals as increasing spore load may inhibit these behaviors more severely.

## Materials and methods

To quantify how infection influences swimming behavior, we used five genotypes of *D. dentifera* collected from four midwestern lakes (Island, Canvasback, Downing, Midland, S1 Table). Clonal lines were raised under standardized conditions for more than three generations to control for maternal effects [30]. For each host genotype, we collected neonates that were less than 24 hours old and raised them individually at 20℃ for 6 days in 50 mL tubes with 45 mL filtered lake water. Every Monday, Wednesday, and Friday, filtered lake water was replaced, and tubes were fed 2 mg C / L of the green algae *Ankistrodesmus falcatus* (Corda) Falfs, 1848 [31]. On day 6, individuals were transferred to 15 mL tubes filled with 10 mL of filtered lake water and 1 mg C / L *A. falcatus*. Individuals assigned to the “infected” treatment received 500 spores / mL of *Australozyma monospora*. To prevent settling of spores, tubes were inverted every hour for a period of five hours on the day of inoculation, and then again the following morning. After 24 hours, all individuals were returned to 50 mL tubes with 45 mL of filtered lake water and 2 mg C / L of *A. falcatus*. Water changes and feeding continued three times a week. Not all *Daphnia* exposed to the fungus will develop an infection due to variation in susceptibility [28]. Therefore, individuals in the “infected” treatment group were checked under a microscope for signs of late-stage infection. Only individuals with a visibly confirmed late-stage infection were used in the “infected” groups in the subsequently described methods.

Starting at 9 days post infection, we recorded videos in the experimental setup described in the next paragraph to capture swimming behavior in infected (only those hosts with a late-stage infection that had been confirmed visually were used) and uninfected individuals. For each genotype, we attempted to measure 3 replicate populations (5 *Daphnia* per population) at three dates post infection: early (day 9 or 10 post infection), middle (day 11 or 12 post infection) and late (day 13 or 14 post infection). These timings were based on the known development time of the fungus in the *Daphnia* host [29]. Given that some genotypes became infected more easily than others and some infected hosts died more often than others, there are cases with only 2 replicate populations per time period (S1 Table). No death occurred during filming. By day 14, there was considerable death among the infected individuals, resulting in no replication for 3 of the 5 clonal lines. Hence, our analysis only includes the first two recording dates (see S1 Fig and S2 Table for full analysis – the results are qualitatively the same except for significance of the interaction between infection status and trial for net distance, which was expected due to the sample size constraints). In total, 255 *Daphnia*, as part of 26 populations each in trials 1 and 2, were included in the analysis of the first two trials.

The experimental set up consisted of 10, 1-m long clear acrylic columns with 3.2-cm outer diameter, 2.9-cm inner diameter, positioned next to a measuring tape for reference. Black sand was added to the bottom to minimize light reflection prior to filling the column with filtered lake water (Merck Millipore Ltd. Glass Fibre Prefilter) to the 75 cm mark. The temperature of the lake water was approximately 20℃. Each acrylic column was a replicate containing 5 *D. dentifera* of the same genotype and infection status. The experiment was run in multiple temporal blocks, with each block consisting of one replicate of each genotype x infection x timepoint combination.

To measure swimming behavior, we used a protocol modified from a 2001 study performed by De Meester [32]. To begin a trial, 5 *D. dentifera* were added to the top of each tube. After two hours in dark conditions, the light source (15-Watt light bulb) was turned on. We recorded videos of each tube (filming four tubes at a time) for the following 10 minutes using a Nikon DS5200 digital camera. After recording, the contents of the acrylic tubes were gently poured through a 35-μm sieve and *Daphnia* were individually returned to 50 mL tubes. The same individuals were filmed again two and four days later. Where there was mortality, individuals were replaced with *Daphnia* of the same genotype, age, and infection status, so that each recorded replicate contained 5 individuals.

### Data analysis

We used the software ImageJ to analyze the video data [33]. Trajectories of the individual *D. dentifera* in each tube were manually tracked using the MTrackJ plug-in. *Daphnia* were recorded for approximately 10 minutes following the light being turned on. The first 5 minutes allowed the *Daphnia* to acclimate to the initial light exposure and was not included in the analysis. The last 5 minutes of the recording was converted into 300 frames which were included in the analysis. Once individual tracks were finalized with the MTrackJ plugin, the ‘Measure’ function in the plugin was used to generate two data sets, one of measurement values for all points and one of measurement values for each track. We used MATLAB to determine the mean depth, speed, total distance (Euclidean distance), and net distance (distance between the first and last positions of the tracking) for each *Daphnia*. Distance is always greater than or equal to net distance and we analyzed both metrics as it is an indication of how the hosts move. For instance, if they swim in a straight line from position A to position B, then the two metrics would be equal. If distance is greater than net distance, then this implies that they move up and down appreciably or that they spiral within the tube.

All downstream analyses of the data generated from MATLAB were performed in R version 4.4.3 [34]. The effect of infection and the time since infection on each of the four response variables (depth, total distance, net distance, and speed) was quantified using linear mixed effects models with the lme4 and lmerTest packages [35,36]. Infection status and time since infection were included as fixed effects in the models. Genotype was included as a random effect, as well as individual *Daphnia* nested in their experimental column. Columns served as the replicates in each trial.

Correlation analysis using measurements for individual *Daphnia* was performed to assess the relationships between each of the response variables: depth, distance, net distance, and speed. We computed a Pearson’s product-moment correlation coefficient for each pair of response variables and evaluated the significance of each correlation.

## Results

**Fig 1:**
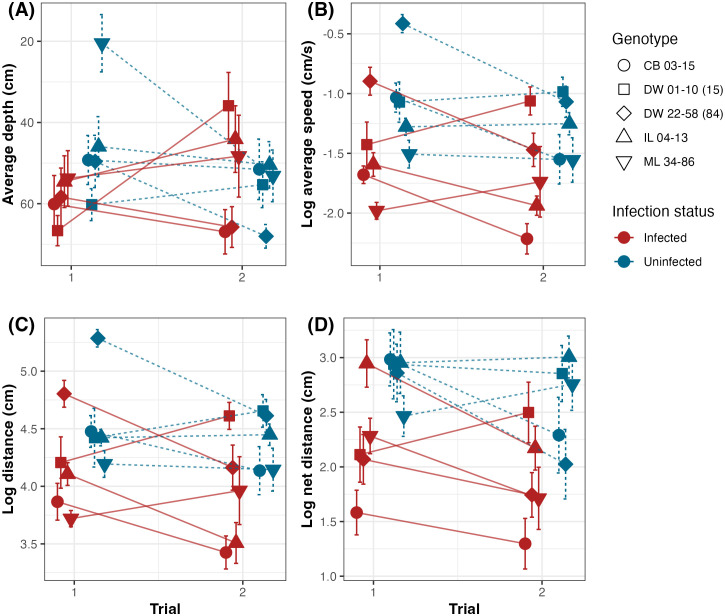
Effect of infection on swimming behavior. Average depth (A), Speed (B), Distance (C), and Net distance (D) were all reduced in infected individuals. Moreover, the differences between infected and uninfected individuals often varied over time. Shapes represent the mean response for each of five *D. dentifera* genotypes across trials, with means for infected and uninfected groups distinguished by color, with red and blue representing infected and uninfected individuals, respectively.

We found an interaction between infection status and time since exposure on average depth (*ꭓ*^2^_1, 25_ = 9.4; p = 0.002; Fig 1A). In trial 2, two infected populations remained near the light source making the average depth of infected populations trend shallower whereas the average depth in the uninfected category trended deeper. Additionally, the average depth decreased substantially for two uninfected populations between trials 1 and 2. These trends are likely responsible for the significant interaction between infection status and time since infection on average depth. The average depth was not influenced by time since exposure (*ꭓ*^2^_1, 25_ = 1.0; *p* = 0.3), but we did find a significant influence of infection (*ꭓ*^2^_1, 25_ = 4.9; *p* = 0.03). Though 50% of the observations fell between 36.7–73.7 cm, average depth ranged from 0.38–74.5 cm. Most individuals, regardless of infection status or trial number, remained near the bottom of the experimental tube (away from the light source).

Swimming speed was strongly influenced by infection status (*ꭓ*^2^_1, 25_ = 48.9; *p* < 0.001) and time since exposure (*ꭓ*^2^_1, 25_ = 12.3; *p* < 0.001; Fig 1B). Infected *D. dentifera* swam more slowly than uninfected individuals (0.25 ± 0.02 and 0.38 ± 0.02 cm/s, respectively). There was no interaction between infection status and time since exposure (*ꭓ*^2^_1, 25_ = 0.03; *p* = 0.9). Swimming speed ranged from 0.04–1.25 cm/s, and the outliers did not significantly affect the output of the model.

This difference in swimming speed translated into significant effects of infection on both total distance (*ꭓ*^2^_1, 25_ = 39.8; *p* < 0.001) and net distance (*ꭓ*^2^_1, 25_ = 40.2; *p* < 0.001) traveled (Fig 1C; 1D). In both cases, the distance traveled was reduced among infected individuals. Across trials, infected *D. dentifera* traveled a shorter total distance than those who were not infected (72.7 ± 4.6 cm and 109.9 ± 5.9 cm, respectively). We observed a similar pattern in net distance, where net distance traveled by infected *D. dentifera* was significantly shorter than uninfected individuals (11.5 ± 1.0 cm and 23.7 ± 1.8 cm, respectively). Time since exposure also significantly impacted the total (*ꭓ*^2^_1, 25_ = 8.2; *p* = 0.004) and net (*ꭓ*^2^_1, 25_ = 7.4; *p* = 0.006) distance traveled, but there was not an interaction between time since exposure and infection status for either total (*ꭓ*^2^_1, 25_ = 0.4; *p* = 0.5) or net (*ꭓ*^2^_1, 25_ = 0.004; *p* = 0.95) distance. The outliers observed among these variables did not significantly affect the output or fit of the model.

**Fig 2:**
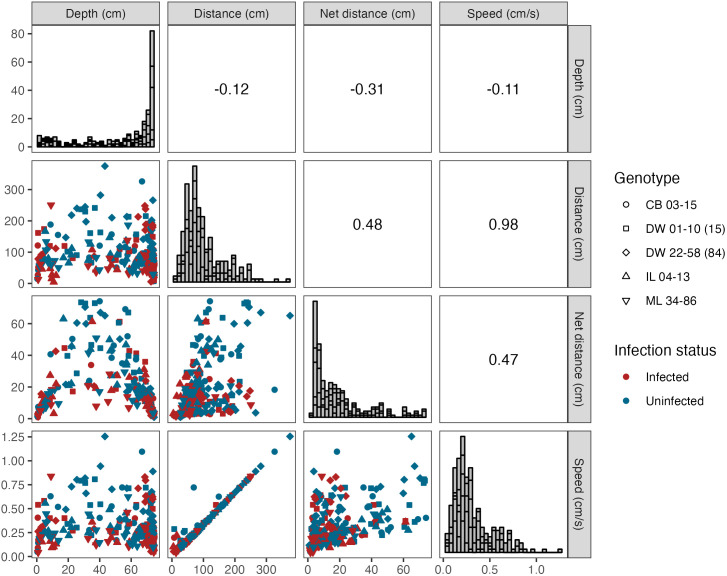
Correlations between measures of swimming behavior. Net distance was negatively associated with depth; individuals who were observed at greater depths traveled a shorter net distance (these individuals remained in the bottom of the tube for most of the trial). Net distance was also positively correlated with distance traveled. Speed is defined as speed = distance/time. Since time is fixed, speed and distance should be perfectly correlated. However, there were eight points that were not perfectly correlated. We believe that in some cases, this is a numerical artifact because some *Daphnia* are stationary for periods of time, and due to human inability to differentiate individuals when their swimming tracks crossed. Color indicates infection status, where red represents infected individuals and blue represents uninfected individuals. Shapes correspond to each of the five genotypes.

Finally, we investigated potential correlations in the four response variables for individual *D. dentifera* and found a significant positive correlation between speed and both total and net distance traveled, as well as between net distance and distance (Fig 2). There was a significant negative association between net distance and depth. Not surprisingly, faster swimmers traveled a greater distance (*r*_255_ = 0.98; *p* < 0.001). Swimming speed is not necessarily predicted to influence net distance if fast swimmers are also the ones who spend more time turning, spiraling, or sinking; however, we did find a correlation between speed and net distance (*r*_255_ = 0.47; *p* < 0.001), likely because individuals that traveled greater total distances also traveled a greater net distance (*r*_255_ = 0.48; *p* < 0.001). Net distance was predictive of the position occupied in the water column (*r*_255_ = −0.31; *p* < 0.001), where individuals that traveled a shorter net distance tended to be found toward the bottom of the water column. However, there was no relationship between position in the water column and total distance traveled (*r*_255_ = −0.12; *p* = 0.06) or swimming speed (*r*_255_= −0.11; *p* = 0.08).

## Discussion

We investigated swimming behavior in five clonal lines of the host species *Daphnia dentifera* in response to infection by its fungal pathogen, *Australozyma monospora.* We found statistical support for the alteration of swimming behavior in infected *D. dentifera.* Average depth, total distance traveled, net distance, and average speed were all significantly reduced in infected individuals compared to uninfected individuals. Contrary to our prediction that time since infection would increase the difference between treatments, we found inconsistent effects of time on the tested variables. While time since exposure did significantly affect swimming speed, total, and net distance, there was no main effect of time on depth selection. However, time since infection did interact with infection status for depth selection. We also found support for a change in phototactic behavior as a result of *A. monospora* infection in *D. dentifera*; the average depth of infected individuals in each trial was significantly different from uninfected individuals. Finally, faster *Daphnia* swam further total and net distances, but swimming speed was not predictive of position in the water column. Only net distance was significantly correlated with depth; there were no correlations between depth and speed, or distance traveled. Our results build on the expanding body of literature exploring the interplay between host behavior and disease.

The observed modifications to swimming behavior of infected *D. dentifera* have implications for changes in habitat selection in natural populations. The reduced distance *D. dentifera* were able to swim, and how fast they were able to do it, could influence the time spent in the warmer surface waters at night, potentially limiting their access to high-quality resources and warmer temperatures that facilitate faster growth and reproduction [37]. Our results are consistent with laboratory assays performed by Johnson and colleagues [20], in which *Daphnia pulicaria* infected with a chytridiomycete exhibited significantly fewer swimming movements. They proposed that altered swimming behavior explains their observations of infected *Daphnia* found at consistently lower depths than uninfected hosts both in field samples and in laboratory assays. Another study [18] found that depth selection of *Daphnia magna* infected with a bacterial pathogen was only altered in light conditions, suggesting potential variation among species and pathogens in changes in diel vertical migration (DVM). Additionally, there is considerable genotypic variation in *Daphnia* populations, as exemplified in our study and others (e.g., [20,32,38,39]). The variation seen in our laboratory study highlights the need to assay multiple clones to increase the relevance to field populations with extensive genetic diversity.

How changes in swimming behavior influence disease transmission is difficult to determine because of multiple opposing forces [40,41]. There are several direct effects of temperature that can alter host-pathogen interactions. As DVM patterns change, the amount of time each day spent in the colder (often below 10℃) and warmer (often above 25℃) water influences not only potential contact rates, but also host growth rate, egg development time, and pathogen growth rate and infectivity. For example, in the *Daphnia magna-Ordospora colligata* Larsson, Ebert & Vávra, 1997 [42] system, a 2022 study found that daily fluctuating temperatures decreased pathogen fitness and restricted its thermal range in terms of both infectivity and within-host pathogen growth; however, a simulated 3-day heat wave did not change infection rates [43]. Another study found temperature effects on transmission of *A. monospora* in that spores grown at lower temperatures were less infective [44]. Changing temperatures not only alter host fitness [45], but changes in lake stratification have also been shown to alter disease transmission dynamics in zooplankton [46,47]. In a 15-year field study, lake stratification was identified as one of the primary drivers of seasonal infection dynamics among *Daphnia pulicaria* populations [48]. In lakes in east-central Illinois, the September temperature in the epilimnion in recent years has exceeded 25℃, whereas earlier records suggest the long-term average was < 20℃ (unpublished data). Simultaneously, earlier ice out and warmer spring temperatures have resulted in early stratification, meaning the bottom water remains a few degrees colder than it had in earlier years (unpublished data). Current research in our group is addressing questions of how changes in stratification patterns may influence disease dynamics.

Changes in swimming and habitat selection behavior such as we observed may also alter interspecific interactions that have both direct and indirect effects on transmission. Because algal resources are non-uniformly distributed in lakes [49], behavioral changes could result in a decreased encounter rate with algal resources. Many questions remain regarding how resources influence host susceptibility to parasites [50,51]. Fewer resources can result in the host having less energy to divert to mounting an immune response, which can increase transmission [39,52]. However, transmission may decrease in food-limited systems if the starving hosts produce fewer infective propagules [53,54]. The changing landscape of resources in lake ecosystems due to anthropogenic change further complicates our understanding of host-pathogen-resource dynamics. Swimming behavior and habitat selection also have implications for predator-prey interactions in terms of encounter (based on habitat selection, and capture) and therefore disease spread. Impaired swimming behavior can increase predation risk by impairing the ability of *Daphnia* to successfully evade their two main predators: fish and the larvae of the phantom midge, *Chaoborus.* Predation by fish removes infected individuals from the population, lowering transmission [14]. In contrast, *Chaoborus* increase transmission through sloppy eating when they consume infected prey [6]; *Daphnia* with impaired swimming may be unable to avoid the rapid *Chaoborus* feeding strike, thereby increasing transmission. Complexity in lake communities demonstrates the difficulty in elucidating disease dynamics in natural populations, and the need for coupling individual behavior with food-web interactions.

## Conclusions and future directions

Although it is clear that infected animals often behave differently than healthy individuals, understanding which parasites and pathogens can actively control the behavior of their hosts, and the mechanisms by which they do so, is still an emerging field [3]. For example, even though several species are known to alter behavior via infection of the host’s central nervous system [55,56], the molecular mechanisms that drive those behavioral modifications are not well resolved. For pathogens of *Daphnia*, we suspect that the behavioral modifications are due to reduced energy, but to our knowledge, the possibility of direct manipulation has not been examined. The research methods developed for insects, such as characterizing any secretions produced by the pathogen, offer a promising research direction [3].

These host-parasite interactions that are rooted in swimming behavior and habitat selection are also influenced by anthropogenic changes. For example, lake ecosystems are increasingly exposed to Artificial Light at Night (ALAN) as urbanization continues [57]. ALAN disrupts natural diel light cycles that influence zooplankton DVM [58–60] and can therefore alter habitat selection and swimming behavior of important grazers such as *Daphnia* [61,62]. Such external disruptions may also alter disease dynamics indirectly (e.g., by impacting host-parasite contact rates) or directly (e.g., by disrupting internal biological processes that impact host physiology and immunity) [63]. Our ongoing research seeks to determine how ALAN and other anthropogenic stressors interact to shape host-parasite interactions and their independent populations.

## Supporting information

S1 Table Number of replicate populations measured in each of the three trials. Each population contained 5 *Daphnia*. Letters indicate late from which the clones were collected (CB = Canvasback, DW = Downing, IL = Island, ML = Midland. The numbers refer to the individual multilocus genotypes determined by microsatellite markers.
(TIFF)

S1 Fig Effect of infection on swimming behavior across all three trials. Average depth (A), Speed (B), Distance (C), and Net distance (D) were all reduced in infected individuals. Moreover, the differences between infected and uninfected individuals often varied over time. Shapes represent the mean response for each of five *D. dentifera* genotypes across trials, with means for infected and uninfected groups distinguished by color, with red and blue representing infected and uninfected individuals, respectively.
(TIF)

S2 Table Results of the ANOVA test on the linear mixed models for average depth, total distance traveled, net distance traveled, and swimming velocity, including data from all trials. The results are qualitatively the same except for significance of the interaction between infection status and trial for average net distance, which was expected due to the sample size constraints.
(TIFF)

## References

[1] Lafferty KD, Morris AK. Altered Behavior of Parasitized Killifish Increases Susceptibility to Predation by Bird Final Hosts. Ecology. 1996;77(5):1390–7. doi: 10.2307/2265536

[2] Berdoy M, Webster JP, Macdonald DW. Fatal attraction in rats infected with Toxoplasma gondii. Proc Biol Sci. 2000;267(1452):1591–4. doi: 10.1098/rspb.2000.1182 11007336 PMC1690701

[3] Libersat F, Kaiser M, Emanuel S. Mind Control: How Parasites Manipulate Cognitive Functions in Their Insect Hosts. Front Psychol. 2018;9:572. doi: 10.3389/fpsyg.2018.00572 29765342 PMC5938628

[4] Behringer DC, Butler MJ, Shields JD. Ecology: avoidance of disease by social lobsters. Nature. 2006;441(7092):421. doi: 10.1038/441421a 16724051

[5] Włodarczyk R, Drzewińska-Chańko J, Kamiński M, Meissner W, Rapczyński J, Janik-Superson K, et al. Stopover habitat selection drives variation in the gut microbiome composition and pathogen acquisition by migrating shorebirds. FEMS Microbiol Ecol. 2024;100(5):fiae040. doi: 10.1093/femsec/fiae040 38515294 PMC11008731

[6] Cáceres CE, Knight CJ, Hall SR. Predator-spreaders: predation can enhance parasite success in a planktonic host-parasite system. Ecology. 2009;90(10):2850–8. doi: 10.1890/08-2154.1 19886493

[7] Duffy MA, Housley JM, Penczykowski RM, Cáceres CE, Hall SR. Unhealthy herds: indirect effects of predators enhance two drivers of disease spread. Funct Ecol. 2011;25:945–53. doi: 10.1111/j.1365-2435.2011.01872.x

[8] Packer C, Holt RD, Hudson PJ, Lafferty KD, Dobson AP. Keeping the herds healthy and alert: implications of predator control for infectious disease. Ecol Lett. 2003;6:797–802. doi: 10.1046/j.1461-0248.2003.00500.x

[9] Adelman JS, Martin LB. Vertebrate sickness behaviors: Adaptive and integrated neuroendocrine immune responses. Integr Comp Biol. 2009;49(3):202–14. doi: 10.1093/icb/icp028 21665814

[10] Altizer S, Bartel R, Han BA. Animal migration and infectious disease risk. Science. 2011;331(6015):296–302. doi: 10.1126/science.1194694 21252339

[11] Stanicka A, Migdalski Ł, Szopieray K, Cichy A, Jermacz Ł, Lombardo P, et al. Invaders as Diluents of the Cercarial Dermatitis Etiological Agent. Pathogens. 2021;10(6):740. doi: 10.3390/pathogens10060740 34208370 PMC8231267

[12] Davenport ES, Dziuba MK, Jacobson LE, Calhoun SK, Monell KJ, Duffy MA. How does parasite environmental transmission stage concentration change before, during, and after disease outbreaks? Ecology. 2024;105(2):e4235. doi: 10.1002/ecy.4235 38185479

[13] Franco SLM, Swenson GJ, Long RA. Year round patchiness of Vibrio vulnificus within a temperate Texas bay. J Appl Microbiol. 2012;112(3):593–604. doi: 10.1111/j.1365-2672.2011.05229.x 22212214 PMC3293253

[14] Hall SR, Duffy MA, Tessier AJ, Cáceres CE. Spatial heterogeneity of daphniid parasitism within lakes. Oecologia. 2005;143(4):635–44. doi: 10.1007/s00442-005-0005-8 15909131

[15] Lampert W. The adaptive significance of diel vertical migration of zooplankton. Funct Ecol. 1989;3:21–7. doi: 10.2307/2389671

[16] Bandara K, Varpe Ø, Wijewardene L, Tverberg V, Eiane K. Two hundred years of zooplankton vertical migration research. Biol Rev Camb Philos Soc. 2021;96(4):1547–89. doi: 10.1111/brv.12715 33942990

[17] Havel JE, Lampert W. Habitat partitioning of native and exotic Daphnia in gradients of temperature and food: mesocosm experiments. Freshw Biol. 2006;51:487–98. doi: 10.1111/j.1365-2427.2006.01511.x

[18] Straus VA. Memoir on the Daphnia, of the class of Crustaceans. Mémoires du Muséum d’Histoire Naturelle. 1820;5:380–425.

[19] Metschnikoff E. Pasteuria ramosa, un microbe parasite des daphnies [Pasteuria ramosa, a microbial parasite of daphnia]. Ann Inst Pasteur. 1888;2:165–70.

[20] Decaestecker E, De Meester L, Ebert D. In deep trouble: habitat selection constrained by multiple enemies in zooplankton. Proc Natl Acad Sci U S A. 2002;99(8):5481–5. doi: 10.1073/pnas.082543099 11960005 PMC122795

[21] Fels D, Lee VA, Ebert D. The impact of microparasites on the vertical distribution of Daphnia magna. Arch Hydrobiol. 2004;161:65–80. doi: 10.1127/0003-9136/2004/0161-0065

[22] Forbes SA. A preliminary report on the aquatic invertebrate fauna of the Yellowstone National Park, Wyoming, and of the Flathead Region of Montana. Bull US Fish Comm. 1893;11:207–58.

[23] Johnson PTJ, Stanton DE, Forshay KJ, Calhoun DM. Vertically challenged: How disease suppresses Daphnia vertical migration behavior. Limnol Oceanogr. 2017;63(2):886–96. doi: 10.1002/lno.10676 32704187 PMC7377221

[24] Sars GO. Om de i Christiania omegn forekommende cladocerer. Forh Vidensk-Selsk Christiania. 1864;1863:18–159.

[25] Metschnikoff E. Ueber eine Sprosspilzkrankheit der Daphnien. Beitrag zur Lehre über den Kampf der Phagocyten gegen Krankheitserreger. Archiv für pathologische Anatomie und Physiologie und für klinische Medicin. 1884;96:177–95. doi: 10.1007/BF02361555

[26] Kamieński F. Notice préliminaire sur la nouvelle espèce de Metschnikowia (Monospora Metschn.). Trav Soc Imp Nat St-Petersbourg. 1899;30:1–4.

[27] Lachance MA, Cáceres CE, Fredericks MJ, Duffy MA, Stewart Merrill TE. Reviving Élie Metschnikoff’s Monospora: the obligately parasitic yeast Australozyma monospora sp. nov. FEMS Yeast Research. 2025;25. doi: 10.1093/femsyr/foaf041 PMC12359140 40824101

[28] Stewart Merrill TE, Hall SR, Merrill L, Cáceres CE. Variation in Immune Defense Shapes Disease Outcomes in Laboratory and Wild Daphnia. Integr Comp Biol. 2019;59(5):1203–19. doi: 10.1093/icb/icz079 31141120

[29] Stewart Merrill TE, Cáceres CE. Within-host complexity of a plankton-parasite interaction. Ecology. 2018;99(12):2864–7. doi: 10.1002/ecy.2483 30265378

[30] Lynch M. The genetic structure of a cyclical parthenogen. Evolution. 1984;38(1):186–203. doi: 10.1111/j.1558-5646.1984.tb00271.x 28556076

[31] Ralfs J. The British Desmidieae. London: Reeve, Benham, and Reeve; 1848. doi: 10.5962/bhl.title.14791

[32] De Meester L. An analysis of the phototactic behaviour of Daphnia magna clones and their sexual descendants. Hydrobiologia. 1991;225(1):217–27. doi: 10.1007/bf00028400

[33] Schneider CA, Rasband WS, Eliceiri KW. NIH Image to ImageJ: 25 years of image analysis. Nat Methods. 2012;9(7):671–5. doi: 10.1038/nmeth.2089 22930834 PMC5554542

[34] RCore Team. R: A language and environment for statistical computing. Vienna, Austria: R Foundation for Statistical Computing; 2025.

[35] Bates D, Maechler M, Bolker B, Walker S. Fitting linear mixed-effects models using lme4. J Stat Softw. 2015;67(1):1–48. doi: 10.18637/jss.v067.i01

[36] Kuznetsova A, Brockhoff PB, Christensen RHB. lmerTest package: Tests in linear mixed effects models. J Stat Softw. 2017;82(13):1–26. doi: 10.18637/jss.v082.i13

[37] Lampert W, McCauley E, Manly BFJ. Trade-offs in the vertical distribution of zooplankton: ideal free distribution with costs? Proc Biol Sci. 2003;270(1516):765–73. doi: 10.1098/rspb.2002.2291 12713752 PMC1691290

[38] Schwarzenberger A, Handke NH, Romer T, Wacker A. Geographic clines in Daphnia magna’s circadian clock gene expression: Local adaptation to photoperiod. Zoology (Jena). 2021;144:125856. doi: 10.1016/j.zool.2020.125856 33202364

[39] Cáceres CE, Stewart Merrill TE. The role of varying resources on Daphnia dentifera immune responses. Fundam Appl Limnol. 2022. doi: 10.1127/fal/2022/1458

[40] Reichwaldt ES, Wolf ID, Stibor H. Effects of a fluctuating temperature regime experienced by Daphnia during diel vertical migration on Daphnia life history parameters. Hydrobiologia. 2005;543(1):199–205. doi: 10.1007/s10750-004-7451-x

[41] Ziarek JJ, Nihongi A, Nagai T, Uttieri M, Strickler JR. Seasonal adaptations of Daphnia pulicaria swimming behaviour: the effect of water temperature. Hydrobiologia. 2010;661(1):317–27. doi: 10.1007/s10750-010-0540-0

[42] Larsson JI, Ebert D, Vávra J. Ultrastructural study and description of Ordospora colligata gen. et sp. nov. (Microspora, Ordosporidae fam. nov.), a new microsporidian parasite of Daphnia magna (Crustacea, Cladocera). Eur J Protistol. 1997;33(4):432–43. doi: 10.1016/S0932-4739(97)80055-7

[43] Kunze C, Luijckx P, Jackson AL, Donohue I. Alternate patterns of temperature variation bring about very different disease outcomes at different mean temperatures. Elife. 2022;11:e72861. doi: 10.7554/eLife.72861 35164901 PMC8846586

[44] Shocket MS, Vergara D, Sickbert AJ, Walsman JM, Strauss AT, Hite JL, et al. Parasite rearing and infection temperatures jointly influence disease transmission and shape seasonality of epidemics. Ecology. 2018;99(9):1975–87. doi: 10.1002/ecy.2430 29920661

[45] Walsh MR, Whittington D, Funkhouser C. Thermal transgenerational plasticity in natural populations of Daphnia. Integr Comp Biol. 2014;54(5):822–9. doi: 10.1093/icb/icu078 24948139

[46] Dallas T, Drake JM. Fluctuating temperatures alter environmental pathogen transmission in a Daphnia-pathogen system. Ecol Evol. 2016;6(21):7931–8. doi: 10.1002/ece3.2539 30128141 PMC6093173

[47] Ozersky T, Nakov T, Hampton SE, Rodenhouse NL, Woo KH, Shchapov K. Hot and sick? Impacts of warming and a parasite on the dominant zooplankter of Lake Baikal. Limnol Oceanogr. 2020;65:2772–86. doi: 10.1002/lno.11550

[48] Johnson PTJ, Ives AR, Lathrop RC, Carpenter SR. Long-term disease dynamics in lakes: causes and consequences of chytrid infections in Daphnia populations. Ecology. 2009;90(1):132–44. doi: 10.1890/07-2071.1 19294920

[49] Winder M, Boersma M, Spaak P. On the cost of vertical migration: are feeding conditions really worse at greater depths? Freshwater Biology. 2003;48:383–93. doi: 10.1046/j.1365-2427.2003.00995.x

[50] Cressler CE, Nelson WA, Day T, McCauley E. Disentangling the interaction among host resources, the immune system and pathogens. Ecol Lett. 2014;17(3):284–93. doi: 10.1111/ele.12229 24350974 PMC4264941

[51] Pike VL, Lythgoe KA, King KC. On the diverse and opposing effects of nutrition on pathogen virulence. Proc Biol Sci. 2019;286(1906):20191220. doi: 10.1098/rspb.2019.1220 31288706 PMC6650706

[52] McKay AF, Ezenwa VO, Altizer S. Consequences of Food Restriction for Immune Defense, Parasite Infection, and Fitness in Monarch Butterflies. Physiol Biochem Zool. 2016;89(5):389–401. doi: 10.1086/687989 27617360

[53] Pulkkinen K, Ebert D. Host starvation decreases parasite load and mean host size in experimental populations. Ecology. 2004;85(3):823–33. doi: 10.1890/03-0185

[54] Civitello DJ, Penczykowski RM, Smith AN, Shocket MS, Duffy MA, Hall SR. Resources, key traits and the size of fungal epidemics in Daphnia populations. J Anim Ecol. 2015;84(4):1010–7. doi: 10.1111/1365-2656.12363 25733032

[55] Bender JA, Pollack AJ, Ritzmann RE. Neural activity in the central complex of the insect brain is linked to locomotor changes. Curr Biol. 2010;20(10):921–6. doi: 10.1016/j.cub.2010.03.054 20451382

[56] Gal R, Libersat F. A wasp manipulates neuronal activity in the sub-esophageal ganglion to decrease the drive for walking in its cockroach prey. PLoS One. 2010;5(4):e10019. doi: 10.1371/journal.pone.0010019 20383324 PMC2850919

[57] Falcón J, Torriglia A, Attia D, Viénot F, Gronfier C, Behar-Cohen F, et al. Exposure to Artificial Light at Night and the Consequences for Flora, Fauna, and Ecosystems. Front Neurosci. 2020;14:602796. doi: 10.3389/fnins.2020.602796 33304237 PMC7701298

[58] Leech DM, Williamson CE. In situ exposure to ultraviolet radiation alters the depth distribution of Daphnia. Limnol Oceanogr. 2001;46(2):416–20. doi: 10.4319/lo.2001.46.2.0416

[59] Leach TH, Williamson CE, Theodore N, Fischer JM, Olson MH. The role of ultraviolet radiation in the diel vertical migration of zooplankton: an experimental test of the transparency-regulator hypothesis. J Plankton Res. 2015;37(5):886–96. doi: 10.1093/plankt/fbv061

[60] Ringelberg J, Flik BJG. Increased phototaxis in the field leads to enhanced diel vertical migration. Limnol Oceanogr. 1994;39:1855–64. doi: 10.4319/lo.1994.39.8.1855

[61] Maszczyk P, Tałanda J, Babkiewicz E, Leniowski K, Urban P. Daphnia depth selection in gradients of light intensity from different artificial sources: An evolutionary trap? Limnology & Oceanography. 2021;66(4):1367–80. doi: 10.1002/lno.11691

[62] Moore MV, Pierce SM, Walsh HM, Kvalvik SK, Lim JD. Verh Internat Verein Limnol. 2000;27(2):779–82. doi: 10.1080/03680770.1998.11901341

[63] Bumgarner JR, Nelson RJ. Light at Night and Disrupted Circadian Rhythms Alter Physiology and Behavior. Integr Comp Biol. 2021;61(3):1160–9. doi: 10.1093/icb/icab017 33787878 PMC8645190

