# Supplementary figures and images for: Swimming behavior of Daphnia dentifera is influenced by Australozyma monospora infection
Source: PLoS One. 2026 May 11;21(5):e0346784. doi: 10.1371/journal.pone.0346784 (PMC13160294; doi:10.1371/journal.pone.0346784)

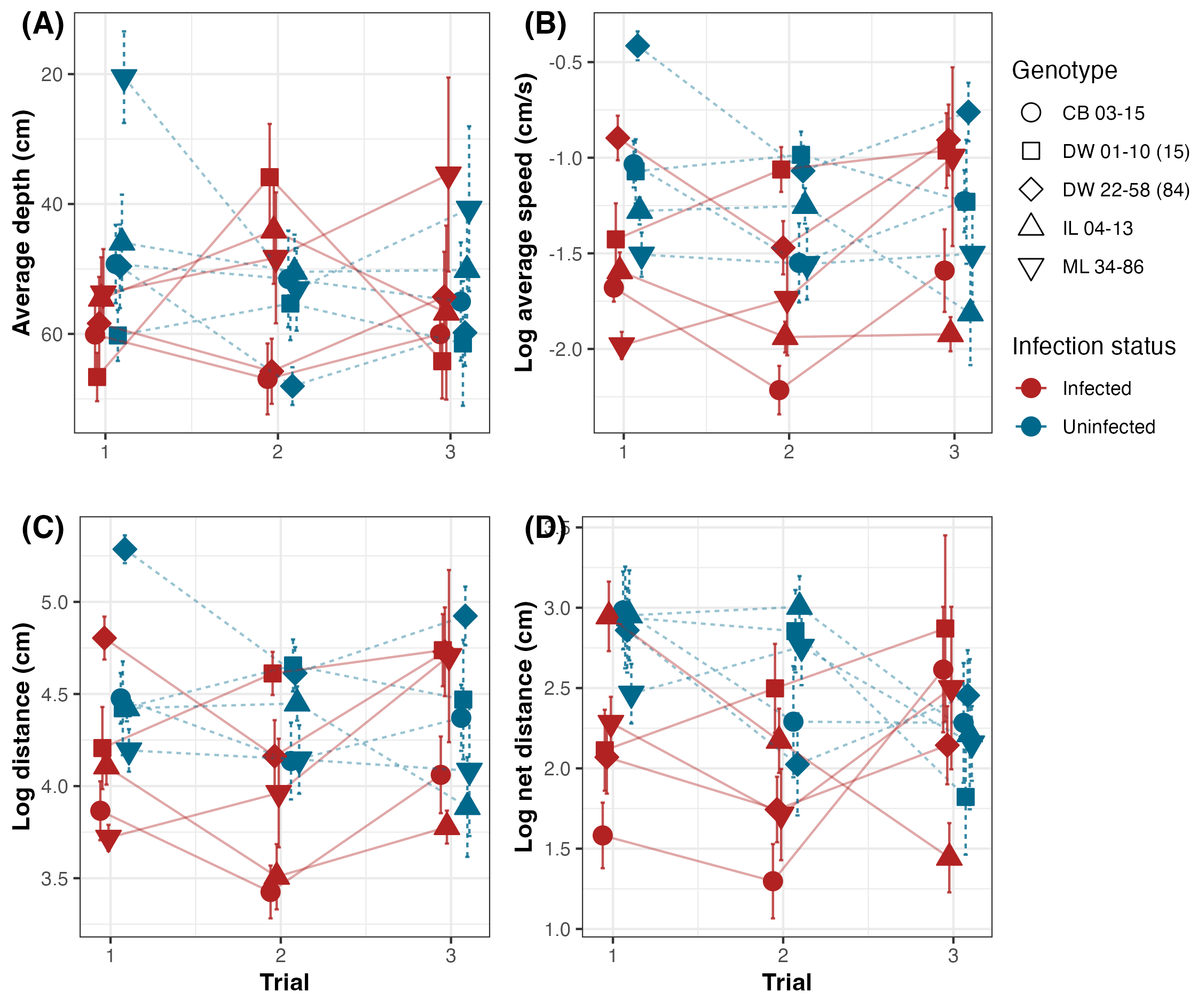

Supplement: S1 Fig — Average depth (A), Speed (B), Distance (C), and Net distance (D) were all reduced in infected individuals. Moreover, the differences between infected and uninfected individuals often varied over time. Shapes represent the mean response for each of five D. dentifera genotypes across trials, with means for infected and uninfected groups distinguished by color, with red and blue representing infected and uninfected individuals, respectively. (TIF) [file pone.0346784.s002.tif]
